# 1*α*,25-Dihydroxyvitamin D3 Supplementation during Pregnancy Is Associated with Allergic Rhinitis in the Offspring by Modulating Immunity

**DOI:** 10.1155/2021/6638119

**Published:** 2021-04-14

**Authors:** Liqing Zhang, Haifeng Ni, Zhen Zhou, Xiaoyang Yuan, Junbo Xia, Bo Jiang, Yong Li

**Affiliations:** ^1^Department of Otolaryngology, Tongxiang First People's Hospital, Tongxiang, Zhejiang 314500, China; ^2^Department of Otolaryngology, Affiliated Hangzhou First People's Hospital, Zhejiang University School of Medicine, Hangzhou, Zhejiang 310006, China

## Abstract

**Background:**

Maternal supplementation with 1*α*,25-dihydroxyvitamin D3 (VD3) has immunologic effects on the developing fetus through multiple pathways. This study was aimed at investigating the effects of VD3 supplementation on immune dysregulation in the offspring during allergic rhinitis.

**Methods:**

Different doses of VD3 as well as control were given to pregnant female mice. Ovalbumin (OVA) challenge and aluminum hydroxide gel in sterile saline were used to induce allergic rhinitis in offspring mice. Nasal lavage fluids (NLF) were collected, and eosinophils were counted in NLF 24 hours after the OVA challenge. Th1, Th2, Th17, and Treg subtype-relevant cytokines, including IFN-*γ*, IL-4, IL-10, IL-17, TGF-*β*, and OVA-IgE levels from the blood and NLF of offspring mice, were detected by the enzyme-linked immunosorbent assay (ELISA) method. The Treg subtype was analyzed by flow cytometry. Treg cells were purified from offspring and were adoptively transferred to OVA-sensitized allogenic offspring mice. The outcomes were assessed in allogenic offspring.

**Results:**

Our data showed that VD3 supplementation significantly decreased the number of eosinophils, basophils, and lymphocytes in the peripheral blood and NLF. The proportion of CD4^+^CD25^+^FoxP3^+^Tregs had a positive correlation with VD3 in a dose-dependent manner. The levels of serum IgE, IL-4, and IL-17 were decreased while the expressions of IFN-*γ*, IL-10, and TGF-*β* were significantly enhanced in VD3 supplementation groups. Adoptive transfer CD4^+^CD25^+^FoxP3^+^Tregs of VD3 supplementation groups promoted Th1 and suppressed Th2 responses in the offspring during allergic rhinitis.

**Conclusion:**

Our findings indicated that low dose VD3 supply in pregnant mice's diet suppressed Th2 and Th17 responses in allergic rhinitis by elevating the Th1 subtype and the proportion of CD4^+^CD25^+^FoxP3^+^Tregs in offspring. It suggested that low dose VD3 supply may have the potential to act as a new therapeutic strategy for allergic rhinitis.

## 1. Introduction

Allergic rhinitis (AR) is one of the most common allergic diseases with progressively increasing prevalence, which affects 36%-40% of children and 10%-30% of adults [1, 2]. AR is a hypersensitivity disease mediated by specific IgE in response to allergens, which is characterized by a destructive balance of Th1 (T helper type 1)/Th2 (T helper type 2) cytokine with Th2 skewing [3]. Although both environmental factors and genetic susceptibility are confirmed to play critical roles in the development of AR [4], its exact pathogenesis remains unknown.

1*α*,25-Dihydroxyvitamin D3 (VD3) was initially characterized for its role in bone metabolism, but recent researches confirmed that it also could regulate the function of various immunocytes and nonimmune cells, including monocytes, dendritic cells, T and B lymphocytes, epithelial cells, and so on [5]. Furthermore, VD3 receptors have also been identified in almost all immunocytes. Most of the immunocytes can also convert vitamin D into VD3 by expressing VD3-activating enzymes [6, 7].

Recent studies have investigated the relationship between the level of VD3 and the incidence of AR [8, 9]. AR incidence was found to decline as VD3 levels increased [10]. A Norwegian study found similar results in women but converse results in men [11]. However, the other study showed that no association between VD3 levels and the incidence of AR has been found [12]. Hence, the relationship between VD3 levels and AR incidences remains unclear, and the role of VD3 in AR appears to be more complex. VD3 deficiency during pregnancy is fairly prevalent in most countries. It was reported that maternal VD3 status during pregnancy may affect the developing immune system of the offspring [13]. For example, a few studies showed that maternal VD3 intake from foods during pregnancy decreased the risk of AR in less than 5-year-old children [14, 15]. However, the other reports showed that intake of VD3 in pregnant women did not have any effects on the incidence of AR in children [16], even increasing the incidence of AR in adulthood [17, 18]. Evidently, the relationship between the VD3 maternal intake during pregnancy and the AR incidence of its offspring should be confirmed by further experiments.

CD4^+^CD25^+^FoxP3^+^Tregs cells have been shown to be critical in the maintenance of immune responses and T cell homeostasis, which suppresses Th2 responses in allergic conditions [19]. Low dose VD3 could promote the proliferation of CD4^+^CD25^+^FoxP3^+^Tregs to induce antigen-specific tolerance in immune-mediated conditions [20]. As we know, naïve T cells can change into different subtypes like Th1, Th2, Th17, and Treg in different culture conditions [21, 22]. Th1, Th2, Th17, and Treg characterize with specific transcription factors and cytokines, which are T-bet and IFN-*γ* for Th1, GATA-3 and IL-4 for Th2, RoR*γ*t and IL-12 for Th17, Foxp3 and CD25 for Treg. Therefore, VD3 supplementation may disrupt the balance of these T lymphocyte subtypes.

Based on these previous studies, we hypothesize that low dose VD3 may suppress Th2 responses to allergens in AR by elevating CD4^+^CD25^+^FoxP3^+^Tregs. In this study, pregnant female mice were supplemented with different low doses of VD3 first; the Th1, Th2, and Th17 responses to allergens in offspring mice were assessed; and the quantity and ability of CD4^+^CD25^+^FoxP3^+^Tregs in offspring mice were detected. The aim of this study was to investigate the optimal dose of VD3 as a supplement for pregnant mice's diet to successfully decrease the allergic rhinitis incidence of their offspring via regulating T lymphocyte functions.

## 2. Methods

### 2.1. Animals and Reagents

A total of 25 eight-week-old female BALB/c mice were purchased from the Animal Resources Centre (Nanjing, China). The animals were raised in a constant-temperature environment at 22 ± 2°C, with a regular 12-hour light/dark cycle. Ovalbumin (OVA) and VD3 were obtained from Sigma (St. Louis, Mo, USA). The enzyme-linked immunosorbent assay (ELISA) kits for mouse VD3, IL-4, IL-10, IFN-*γ*, IL-17, TGF-*β*, and OVA-IgE were obtained from Abcam (Cambridge, MA, USA). Mouse Treg Flow Kit (FOXP3 Alexa Fluor® 488/CD4 APC/CD25 PE) was purchased from BioLegend, San Diego, CA.

### 2.2. Mating, Parturition, Offspring, and Preparation of Animal Model

The female mice were allowed to mate with males at a 2 : 1 ratio, and mating was confirmed by observing the vaginal smear. Female mice showing sperm in the smear were separated on the day of detection, and this was considered as gestation day (GD) 0. The day on which mice were born before 4:00 pm was designated as postnatal day (PND) 0. Offspring mice remained with their mothers until weaned at PND 21. Twenty-five pregnant female mice were randomly divided into 5 groups (*n* = 5). The control group received subcutaneous injections of 1 mL of 0.9% saline; the 50 ng, 100 ng, 150 ng, and 200 ng VD3 groups were correspondingly given 50 ng, 100 ng, 150 ng, and 200 ng (diluting to 1 mL used 0.9% saline, equal to 100, 200, 300, and 400 IU/kg/daily) VD3 by subcutaneous injections once a day during pregnancy. Blood was obtained from the pregnant female mice to test the level of VD3 at GD 0, 1, 12, and 25.

### 2.3. Sensitization and Challenge for Offspring Mice

To establish allergic rhinitis mouse models, six-week-old offspring were first sensitized with an intraperitoneal injection of 50 *μ*g OVA and 5 mg aluminum hydroxide gel in sterile saline from days 0 to 2. After systemic sensitization, mice were locally challenged by intranasal instillation with 50 *μ*g OVA and 50 *μ*L phosphate-buffered saline (PBS) into their nostrils from day 7 to day 12. After the last challenge, frequency of sneezing and nose rubbing behavior was recorded for at least 15 min.

### 2.4. Eosinophil Counts in Nasal Lavage Fluid

Nasal lavage fluids (NLF) were collected 24 hours after the last intranasal challenge with OVA through a pulmonary lavage technique with sterile saline. Total cell numbers in NLF were determined in duplicates with a hemocytometer. Eosinophils were stained with the Wright-Giemsa and 500 cells were counted in total from each sample at a magnification of ×200 (oil immersion).

### 2.5. Serum OVA-Specific IgE and Cytokine Detection in Peripheral Blood and Nasal Mucosa Tissues

Blood (0.1 mL) and nasal mucosa tissue were collected from offspring mice. The serum was obtained from blood by centrifugation at 3000 revolutions per minute (rpm) for 10 minutes at 4°C. The nasal mucosa was homogenated and centrifuged at 3000 rpm for 10 minutes at 4°C, and the supernatants were collected for cytokine detection. The levels of IL-4, IL-10, IFN-*γ*, and OVA-IgE in peripheral blood and nasal mucosa tissues were assessed by an ELISA kit.

### 2.6. The Analysis of CD4^+^CD25^+^FoxP3^+^Tregs by Flow Cytometry

The flow cytometry assay was carried out according to the manufacturer's guidelines. Briefly, spleens from offspring were processed to prepare splenocytes. A Mouse Treg Flow Kit was used to double stain the collected and enriched CD4^+^T cells with fluorescence-conjugated antibodies (CD25-phycoerythrin and CD4-allophycocyanin); then, the cells were stained with Alexa Fluor 488-conjugated FoxP3 antibody. CD4^+^CD25^+^FoxP3^+^Tregs were analyzed by flow cytometry.

### 2.7. Adoptive Transfer of CD4^+^CD25^+^FoxP3^+^Tregs into OVA-Sensitized Allogenic Offspring

Briefly, CD4^+^CD25^+^FoxP3^+^Tregs were purified from offspring in each group and 5 × 10^4^ cells were resuspended in 0.2 mL 0.9% saline and then were adoptively transferred through the tail vein of a corresponding group of OVA-sensitized allogenic offspring. 24 hours later, the same outcomes were assessed in allogenic offspring.

### 2.8. Statistical Analysis

All statistical analyses were performed using the GraphPad Prism 5.0 (San Diego, CA, USA) and SPSS 21.0 version (Chicago, IBM Inc., USA). Data are expressed as the mean ± SEM. Statistical significance of the pregnancy supplementation days and level of serum VD3 administration was calculated using two-way ANOVA with Bonferroni post hoc tests. Independent-sample *T* test was used to compare between each VD3 group and the control group. Pearson's correlation analysis was used to determine if there was a correlation between the aforementioned parameters. ^∗^*P* < 0.05, ^∗∗^*P* < 0.01, and ^∗∗∗^*P* < 0.001 were considered statistically significant.

### 2.9. Ethics Approval and Consent to Participate

All animal care and experimental protocols were approved by the Institutional Animal Care and Use Committee (INHA 150309-351-2). The study was approved by the local ethics committee of Hangzhou First People's Hospital, China (approval ID: 201710701).

## 3. Results

### 3.1. Serum VD3 levels of Pregnant Mice

**Figure 1:**
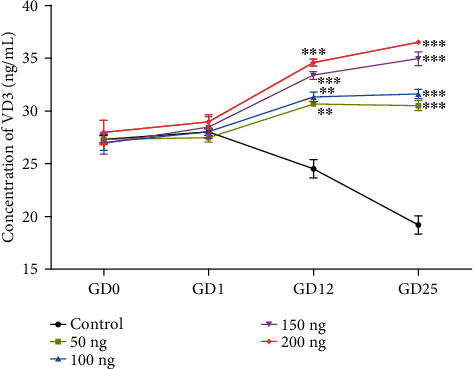
The response of the serum VD3 levels of pregnant mice to different doses of VD3. There was a positive dose-dependent manner between the supplementation time and the concentration of VD3. Values are mean ± SEM (*n* = 5). GD: gestation day. ^∗∗^*P* < 0.01 vs. control and ^∗∗∗^*P* < 0.001 vs. control.

The serum VD3 levels in pregnant mice after different doses of VD3 injection are illustrated in Figure 1. We found that the VD3 levels of pregnant mice were the same baseline at GD0 and GD1. During pregnancy, the VD3 concentration persistently decreased in the control group; however, it stably increased in all VD3 groups. Compared with the control group, the serum VD3 levels in the VD3 group had no significant difference at GD0 and GD1. However, significant differences were found at GD12 and GD25. Furthermore, the levels of VD3 in pregnant mice were increased in a supplementation time and the concentration in a VD3-dependent manner.

### 3.2. VD3 Maternal Supplementation Influenced Immune Response of the Offspring during AR

**Figure 2:**
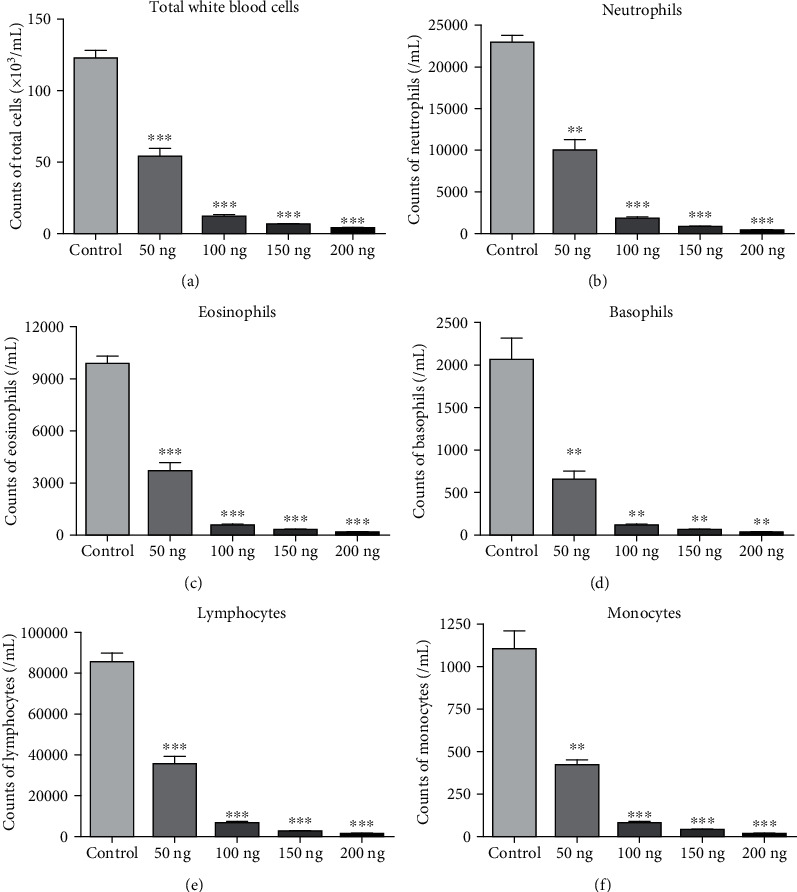
Immune cells were counted in offspring mice from VD3-treated group: (a) total white blood cells, (b) neutrophils, (c) eosinophils, (d) basophils, (e) lymphocytes, and (f) monocytes were counted by mouse cell count machine from peripheral blood of offspring mice. ^∗^*P* < 0.05 VD3 treatment vs. control; ^∗∗^*P* < 0.01 VD3 treatment vs. control; ^∗∗∗^*P* < 0.001 VD3 treatment vs. control.

**Figure 3:**
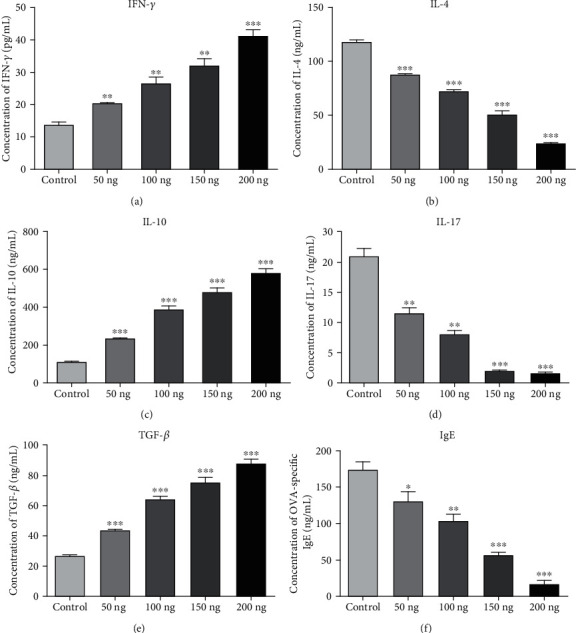
Cytokines were measured by ELISA in offspring mice from VD3-treated groups: (a) IFN-*γ*, (b) IL-4, (c) IL-10, (d) IL-17, (e) TGF-*β*, and (f) IgE from peripheral blood of offspring mice in VD3-treated and control groups were measured by ELISA. ^∗^*P* < 0.05 vs. control, ^∗∗^*P* < 0.01 vs. control, and ^∗∗∗^*P* < 0.001 vs. control.

To investigate the effects of VD3 maternal supplementation on immune response, we measured immune cell profiles in the peripheral blood of offspring mice. The results are shown in Figures 2(a)–2(f). Total white blood cells, neutrophils, eosinophils, basophils, lymphocytes, and monocytes were significantly decreased in a VD3 dose-dependent manner. We further detected the levels of IFN-*γ*, IL-4, IL-10, IL-17, TGF-*β*, and IgE in peripheral blood by ELISA (Figure 3). It was found that the levels of IFN-*γ*, IL-10, and TGF-*β* in the peripheral blood of offspring mice were dramatically elevated with VD3 administration (Figures 3(a), 3(c), and 3(e)). In contrast, IL-4 and IL-17 levels were markedly declined in a VD3 dose-dependent manner (Figures 3(b) and 3(d)). We also found that there was a positive correlation between the IgE and IL-4 levels in the peripheral blood with a VD3 supplementation dose (Figure 3(f)).

### 3.3. The Proportion of CD4^+^CD25^+^FoxP3^+^Tregs in VD3 Treated Groups from Offspring by Flow Cytometry

**Figure 4:**
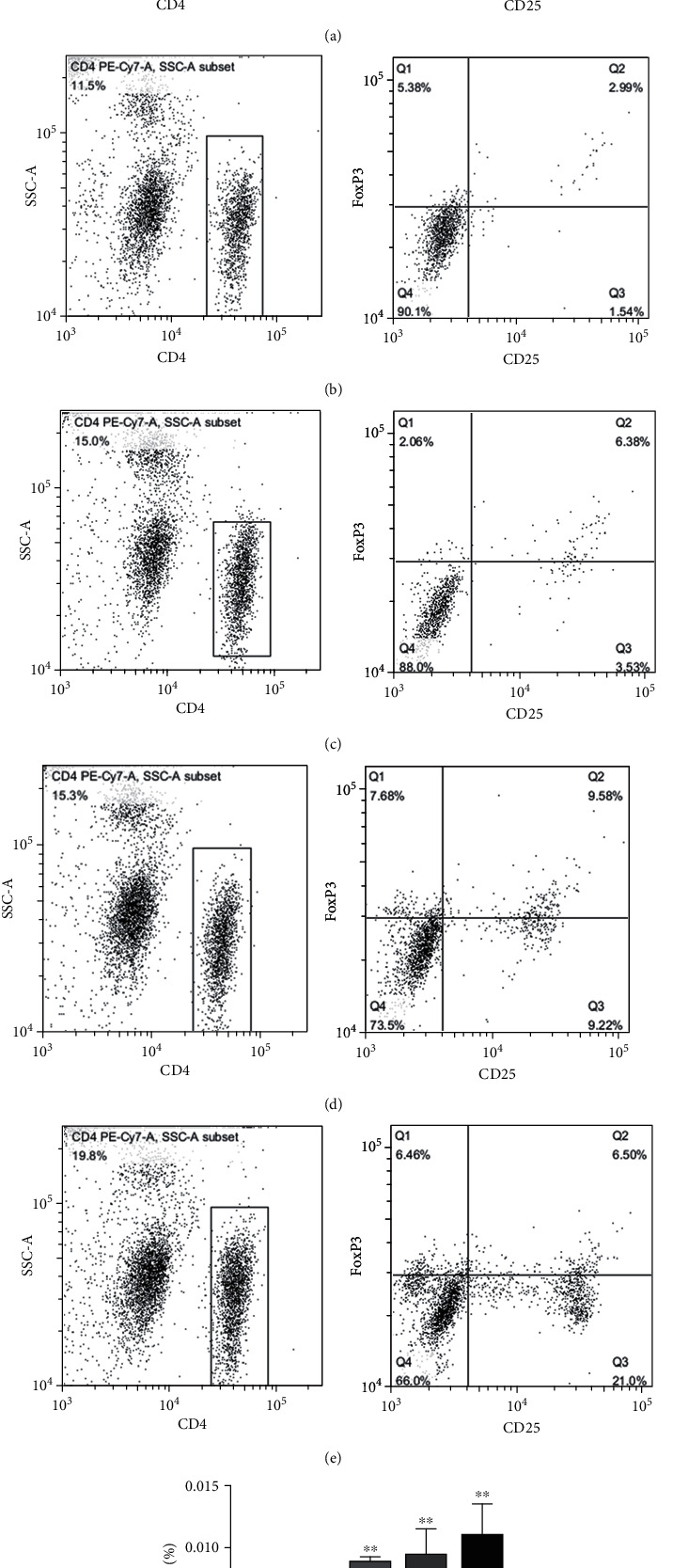
The proportion of CD4^+^CD25^+^FoxP3^+^Tregs in VD3 groups from offspring. Compared with control group (a), the proportion of CD4^+^CD25^+^FoxP3^+^Tregs was elevated in VD3 groups ((b) 50 ng, (c) 100 ng, (d) 150 ng, and (e) 200 ng groups) from offspring in a dose dependency (f). Values are mean ± SEM (*n* = 5), ^∗^*P* < 0.05 vs. control and ^∗∗^*P* < 0.01 vs. control.

In addition, we also analyzed CD4^+^CD25^+^FoxP3^+^Tregs by flow cytometry. Compared with the control group, the proportion of CD4^+^CD25^+^FoxP3^+^Tregs was elevated (Figures 4(a)–4(e)). There was also a positive correlation between the CD4^+^CD25^+^FoxP3^+^Tregs proportion and the VD3 supplementation dose (Figure 4(f)).

### 3.4. Adoptive Transfer CD4^+^CD25^+^FoxP3^+^Tregs from Offspring of VD3 Groups Reduce Serum IgE, Th2, and IL-17 Cytokine and Increase Th1 Cytokine in OVA-Sensitized Allogenic Offspring

**Figure 5:**
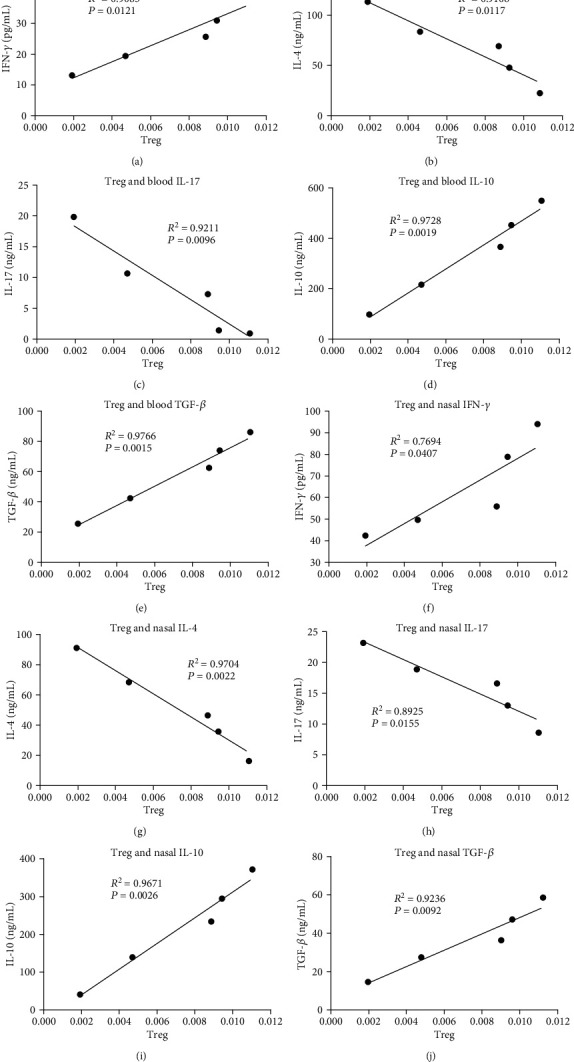
The correlation analyses of cytokine levels and the Treg cell number were analyzed in allogenic offspring after transferring CD4^+^CD25^+^FoxP3^+^Tregs from offspring in VD3 groups. (a) Treg and IFN-*γ*, (b) Treg and IL-4, (c) Treg and IL-17, (d) Treg and IL-10, and (e) Treg and TGF-*β* from peripheral blood of offspring mice with VD3 administration were analyzed for their correlation. (f) Treg and IFN-*γ*, (g) Treg and IL-4, (h) Treg and IL-17, (i) Treg and IL-10, and (j) Treg and TGF-*β* from NLF of offspring mice with VD3 administration were analyzed for their correlation.

**Figure 6:**
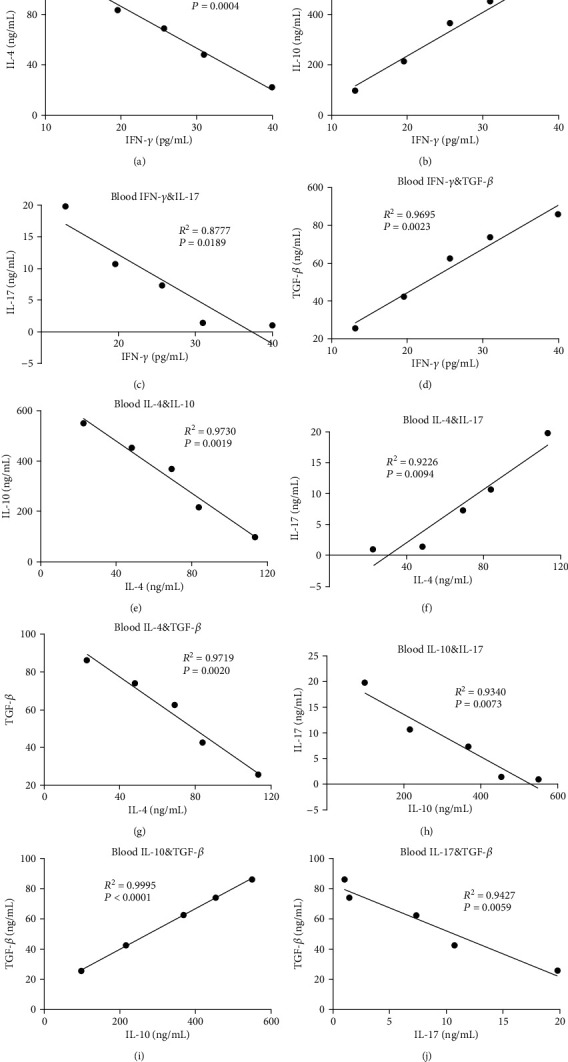
The correlation analyses of cytokines from peripheral blood were analyzed in allogenic offspring after transferring CD4^+^CD25^+^FoxP3^+^Tregs: (a) IFN-*γ* and IL-4, (b) IFN-*γ* and IL-10, (c) IFN-*γ* and IL-17, (d) IFN-*γ* and TGF-*β*, (e) IL-4 and IL-10, (f) IL-4 and IL-17, (g) IL-4 and TGF-*β*, (h) IL-10 and IL-17, (i) IL-10 and TGF-*β*, and (j) TGF-*β* and IL-17 from peripheral blood of offspring mice with Treg cells transferred in VD3 administration were analyzed for their correlation.

To investigate whether the effects of vitamin D3 maternal supplementation on immune regulation depends on Treg cells, we transferred CD4^+^CD25^+^FoxP3^+^Tregs from VD3 groups to OVA-sensitized allogenic offspring. After the adoptive transfer of CD4^+^CD25^+^FoxP3^+^Tregs to allogenic offspring, there was a positive correlation between the number of Tregs and IFN-*γ* or IL-10 or TGF-*β* in either the peripheral blood (Figures 5(a), 5(d), and 5(e)) or NLF (Figures 5(f), 5(i), and 5(j)) from the VD3 group compared with the control group. In contrast, the levels of IL-4 and IL-17 in the peripheral blood (Figures 5(b) and 5(c)) and NLF (Figures 5(g) and 5(h)) had a negative correlation with the adoptively transferred Treg cell number increase in VD3-treated groups compared with the control group. We further explored the relationship among IFN-*γ*, IL-4, IL-10, IL-17, and TGF-*β* cytokines in a Treg cell adoptive transfer (Figure 6). The results showed that the IL-4 and IL-17 levels with IFN-*γ* had a negative correlation (Figures 6(a) and 6(c)) in Treg cell adoptive transferred groups in VD3-treated mice. In contrast, there was a positive correlation between IFN-*γ* and IL-10 or TGF-*β* (Figures 6(b) and 6(d)). We also found that there was a negative correlation between IL-4 and IL-10 (Figure 6(e)), IL-4 and TGF-*β* (Figure 6(g)), IL-10 and IL-17 (Figure 6(h)), and TGF-*β* and IL-17 (Figure 6(j)) in Treg-transferred mice. There was a positive correlation between IL-4 and IL-17 (Figure 6(f)) and IL-10 and TGF-*β* (Figure 6(i)). The results indicated that Treg cell transfer promoted Th1, but inhibited Th2 and Th17 cell development.

## 4. Discussion

Although the associations between VD3 and clinical outcomes have been identified, there has been much less progress in elucidating the underlying *in vivo* biologic mechanism for this association. In this study, we found that VD3 was gradually decreased in the control group during pregnancy, while VD3 was gradually increased in all VD3 groups. This phenomenon was dose-dependent on the supplementation time and the concentration of VD3. Thus, maternal VD3 supplementation during pregnancy could remarkably increase serum VD3 levels.

Several studies *in vitro* found that VD3 could directly promote mature B-cell apoptosis [7]. Furthermore, VD3 also could inhibit antigen-specific B-cell proliferation and antibody secretion. Milovanovic et al. demonstrated that VD3 inhibited IgE production by B-cell [23]. Another study also reported that VD3 supplementation at weaning significantly reduced the serum IgE levels [13]. In our study, we also confirmed that the levels of serum IgE in the peripheral blood were significantly decreased in the offspring of the VD3 group compared with the control group. On the contrary, Matheu et al. reported that IgE production was increased in allergic airway disease mice that were treated early with VD3 [24]. Interestingly, Drozdenko et al. found that there were no significant differences in the frequencies of antibody secreting plasma cells and serum immunoglobulin concentrations in humans after VD3 intake [25]. Similarly, our results also found that maternal VD3 supply during pregnancy significantly decreased serum IgE levels of the offspring in a dose-dependent manner. Furthermore, serum IgE levels have a positive correlation with IL-4 and IL-17 levels and a negative correlation with IL-10, TGF-*β*, and IFN-*γ* levels in offspring. As we know, IFN-*γ*, IL-4, and IL-17 are secreted from Th1, Th2, Th17, respectively [26, 27]. Our result revealed that VD3 might promote Th1, but inhibited Th2 and Th17 cell development.

It was reported that VD3 could recruit eosinophils to noninflammatory sites by regulating C-X-C chemokine receptor type 4 expression [28]. El-Shazly and Lefebvre demonstrated that VD3 modulated a novel inflammatory crosstalk between NK cells and eosinophils via the IL-15/IL-8 axis [29]. Yip et al. also confirmed that VD3 regulated mast cell function and played an anti-inflammatory effect *in vitro* and *in vivo* [30]. Matheu et al. reported that early treatment with VD3 significantly ameliorated eosinophil infiltration in bronchoalveolar lavage fluid and lung tissue with inferior levels of IL-5 [24]. Another study also reported that VD3 supply at weaning significantly reduced pulmonary eosinophilia [13]. In our study, we also confirmed that maternal VD3 supply during pregnancy significantly decreased eosinophils, neutrophils, basophils, lymphocytes, and monocytes in the peripheral blood and NLF of the offspring in a dose-dependent manner with statistical difference. This result indicated that VD3 administration can significantly decrease IgE secreting cells.

The study showed a clear efficacy of the food supplement VD3 in the relief of the signs and symptoms of seasonal allergic rhinitis and in the reduction of the consumption of antiallergic drugs [31]. Compared with sensitized mice without specific immunotherapy or specific immunotherapy alone, the levels of IL-4 were reduced by VD3 plus specific immunotherapy [32]. Perinatal VD3 deficiency alone promoted Th2 skewing and reduced IL-10-secreting T regulatory cells. Furthermore, perinatal VD3 deficiency contributed to asthma severity with worse eosinophilic inflammation and airway remodeling in neonates. Importantly, supplementation with VD3 improves both of these pathological abnormalities [13]. In our study, the same results were found wherein maternal VD3 supply during pregnancy significantly increased Th1 cytokine IFN-*γ* levels and reduced Th2 cytokine IL-4 and Th17 cytokine IL-17 levels of the offspring in a dose-dependent manner. Ozkara et al. reported similar results that VD3 levels showed a negative correlation with IL-4 levels and a positive correlation with IFN-*γ* levels in nasal polyposis patients together with allergic rhinitis [33]. CD4^+^CD25^+^FoxP3^+^Tregs have been confirmed to play a pivotal role in allergic diseases such as asthma, hay fever, and allergic rhinitis [34]. CD4^+^CD25^+^FoxP3^+^Tregs were capable of suppressing Th2 responses to allergens in allergic conditions [35]. The positive correlation between VD3 and Foxp3^+^Tregs in the airways was observed in a severe pediatric asthma cohort. Equally, *in vitro* VD3 could selectively expand Foxp3^+^Tregs [20]. On the other hand, VD3-induced IL-10 production by T cells and IL-10 together with VD3 also acted as a positive autocrine factor for further IL-10 production [36]. VD3 also promotes regulatory immune pathways via generation of tolerogenic antigen presenting cells and FoxP3^+^Tregs or IL-10 [37]. In our study, we confirmed that *in vivo* absolute numbers of CD4^+^CD25^+^FoxP3^+^Tregs in the offspring from pregnant mice with different doses of VD3 were remarkably elevated in a dose-dependent manner. Furthermore, the proportion of CD4^+^CD25^+^FoxP3^+^Tregs has a positive correlation with IL-10, TGF-*β*, and IFN-*γ* levels, and a negative correlation with IL-4, IL-17, and IgE levels in offspring. In addition, we examined the expression of cytokines in the allogenic offspring after adoptively transferring CD4^+^CD25^+^FoxP3^+^Tregs cells from VD3 supplementation offspring. Our results indicated that VD3 strengthens the ability of CD4^+^CD25^+^FoxP3^+^Tregs to suppress Th2 and Th17 responses and enhance Th1 responses. These results further clarified that the effects of VD3 supplementation during pregnancy on immune regulation was dependent on CD4^+^CD25^+^FoxP3^+^Tregs. Our results were consistent with the results of Gorman and Vijayendra reports *in vitro* and *in vivo* [38]. The present study indicated that low dose VD3 supplementation during pregnancy might suppress Th2 responses and enhance Th1 responses to OVA-induced AR by elevating the absolute numbers and strengthening the ability of CD4^+^CD25^+^FoxP3^+^Tregs in offspring. It is suggested that VD3 supplementation or expansion of allergen-specific CD4^+^CD25^+^FoxP3^+^Tregs has the potential to be a new therapeutic strategy for AR.

## 5. Conclusion

Our results showed the relationship between VD3 and the immune system; VD3 and allergic diseases *in vivo* were inconsistent with those *in vitro*. The ability of VD3 supplementation to result in clinical improvement may vary with the dosing regimen or have different effects on different populations. VD3 can significantly elevate the Treg cell number and might promote Th1 but inhibit Th2 and Th17 cell production.

## Data Availability

All data collection and analysis were conducted under double blind and were supported by the Affiliated Hangzhou First People's Hospital, Zhejiang University School of Medicine. We will provide the original data at any time if necessary. Dr. Haifeng Ni and Dr. Yong Li are responsible for the data.
